# The sGC stimulator BAY 41-8543 in a rat model of hypertension-induced heart failure

**DOI:** 10.1186/2050-6511-16-S1-A57

**Published:** 2015-09-02

**Authors:** N Haase, N Wilck, L Marko, A Balogh, A Heuser, D Brockschnieder, A Kretschmer, J P Stasch, N Müller, R Dechend

**Affiliations:** 1 Experimental and Clinical Research Ctr., Max-Delbrueck Ctr. & Medical Faculty of Charite, Berlin, Germany; 2 Max-Delbrueck Ctr., Berlin, Germany; 3 Bayer HealthCare, Global Drug Discovery, Wuppertal, Germany; 4 Helios Klinik, Berlin, Germany

## 

Hypertension with left ventricular hypertrophy is a major cause of diastolic heart failure (DHF). Due to its high prevalence and high rate of mortality, DHF represents a major challenge in today's cardiovascular medicine; with limited therapeutic options. Soluble guanylate cyclase (sGC) stimulation is emerging as a promising treatment option in DHF, and is currently under investigation in preclinical and clinical studies. The present study investigates the effect of the sGC stimulator BAY 41-8543 in a transgenic rat model of hypertension-induced heart failure.

We used 4 week-old male double transgenic rats expression both human renin and angiotensinogen genes (dTGRs). At 7 weeks of age, dTGRs exhibit striking cardiac hypertrophy with fibrosis and inflammation, ventricular arrhythmias and heart failure, which is accompanied with high mortality. We compared vehicle-treated dTGR (receiving 10% transcutol, 20% cremophor, 70% water) to those receiving 3 mg/kg/d BAY 41-8543, and vehicle-treated SD control rats (single oral dose per day for 3 weeks). We performed in vivo echocardiography, hemodynamic monitoring, cardiac electrophysiology studies and blood pressure measurements. Endothelial function was measured in isolated mesenteric arteries. Transcriptional analyses in cardiac tissue were performed using qRT-PCR and gene-microarray. Cardiac tissue was analyzed using histology.

Treatment of dTGRs with BAY 41-8543 resulted in 100% survival at week 7, whereas only 24% of vehicle-treated dTGRs survived. Mean arterial pressure in dTGRs was significantly by BAY 41-8543 reduced (197 ± 11 mmHg vehicle vs 133 ± 4 mmHg BAY 41-8543). In addition, BAY 41-8543 significantly decreased in vivo total peripheral resistance and improved endothelium-dependent vasorelaxation of isolated mesenteric arteries. Furthermore BAY 41-8543 prevented fibrosis and inflammation of cardiac tissue. Echocardiography and invasive hemodynamic monitoring revealed BAY 41-8543 significantly increased ejection fraction and cardiac output in dTGR, whereas vehicle-treated had preserved systolic function but reduced diastolic function. In addition, diastolic compliance was significantly enhanced by BAY 41-8543, as shown by myocardial strain analysis and end-diastolic pressure volume relationship (EDPVR); indicative of an improved diastolic function. In vivo programmed electrical stimulation revealed a high ventricular tachycardia induction rate in vehicle-treated dTGRs (46%), which was significantly reduced in BAY 41-8543-treated dTGR (11%). Myocardial gene-microarray analysis showed a reversal of dysregulated genes in dTGR by BAY 41-8543 treatment.

Our data demonstrate that BAY 41-8543 improves survival and cardiac performance in a transgenic rat model of hypertension-induced DHF. We postulate that treatment of DHF with sGC stimulators offers a novel therapeutic potential for humans.

